# Esophageal papillomatosis: an exceedingly rare disease

**DOI:** 10.1186/s13023-023-02703-8

**Published:** 2023-04-29

**Authors:** Dandan Li, Changfeng Li, Yuxing Yan, Minya Liu

**Affiliations:** Department of Gastrointestinal Medicine (Endoscopy Center), Jilin University, China-Japan Union Hospital, 126 Xiantai Street, Erdao, Changchun, 130033 People’s Republic of China

**Keywords:** Esophageal papillomatosis, Human papilloma virus, Endoscopy, Esophageal cancer, Treatment

## Abstract

If esophageal papilloma (EP) is a rare condition, esophageal papillomatosis (EPS) is a distinct rarity. To date, only 53 well documented cases have been described in English literature. However, the number of reports on EPS significantly increased to over 40 cases during the past 20 years. Perhaps, this is due to the broad use of endoscopy and related research achievements. Most of the cases are individual and it seems that there are no associations between them. And up to now no guidelines can be followed. To further understand this exceedingly rare disease, we had a comprehensive review of the epidemiology, etiology, clinical manifestations, pathogenesis, treatment, and clinical course of EPS.

## Introduction

The criteria of EP were first demonstrated by Stout and Lattes in the 1950s [1, 2]. EP was defined as a coral-like sessile lesion that has a central core of fibrovascular tissue, capped by a squamous epithelium. Adler et al. [3] described the first case of EP that was histologically confirmed. EP prevalence varies from 0.007 to 0.45% [4, 5] and solitary EP is often observed under endoscopy. Occasionally, several EP in one case can also be reported; however, multiple, or extensive EP is extremely rare. In 1977, the first EPS case was inadvertently found in a 3 1/2-year-old boy, who was diagnosed with papilloma of the pyriform sinus, blocking the supraglottis [6]. In this report, multiple EP was first described as EPS.

Most studies thought of EPS as multiple and extensive esophageal squamous papillomas. So far, there are only 53 cases that can be found in English literature, and that are reported in a form of case reports or combined with mini reviews. Although considered as part of EP, EPS has its own characteristics. In this review, we provide an overview of the case studies and summarize the epidemiology, etiology, clinical manifestation and pathology, therapy, and prognosis of EPS.

## Epidemiology of EPS

EPS can be found at any age, from 3.5 months to 91-year-old [7, 8] and the average onset age is approximatively 46.8 years. No significant differences were observed between affected men (24 cases) and women (29 cases). Most of the cases are sporadic and some studies showed that the EP geographic distribution is considerably different [9, 10]. However, based on the literature, there is no evidence that EPS is associated with a geographic origin.

## Etiology of EPS

Although the etiology of EPS is as unclear as EP, it is now thought that several factors are related to EPS incidence, such as chronic mucosal irritation, human papilloma virus (HPV) and genetic factors. It seems similar to EP but has its own characteristics.

### Chronic mucosal irritation

EPS may result from chronic mucosal irritation which is induced by chemical and mechanical mucosal injuries. Gastroesophageal reflux is the most popular chemical factor that is associated with an underlying esophageal inflammatory process. In animal model studies, gastroesophageal and duodenal reflux have been confirmed to induce EPS [11–13]. Among the EPS cases, more than a quarter of patients had a prolonged chronic reflux disease and repeated heartburn. Chemical induction experiments were also performed using the carcinogen, diethylnitrosamine (DEN) [13] and EPS was detected in approximatively 50% of the rats that were fed with DEN alone and this proportion increased to 61.1% when associated with gastroesophageal reflux. Progressive hyperplasia of the papillae and epithelium led to the formation of papillomas. Other chemical factors, such as tobacco and alcohol abuse are also considered as a lifetime risk of EP [10, 14], which can also be found in EPS cases.

Some reports suggested that mechanical injuries, such as previous and repeated esophageal dilatations, a prolonged nasogastric intubation, and metal stent, were associated with the occurrence of EP [10]. It can also been seen in EPS cases [15–17]. For instance, a 78-year-old man with distal esophagus stricture, who had to undergo repeated esophageal dilatations every 6–8 weeks and for more than 4 years, was diagnosed with EPS [16]. Another example was reported by Karras [17] who described the first EPS case following a 6-week placement of a self-expanding metal stent in a patient.

All the above conditions support the hypothesis that mucosal injury and regeneration underlie the etiology of EPS formation. A cellular damage-repair response, induced by prolonged chemical and mechanical mucosal irritations could contribute to an hyperregeneration process, associated with EPS development.

### HPV

HPV is now considered to be closely related to EP and esophageal cancer and was suggested as one of the etiological factors [18, 19]. HPV is a non-enveloped double-stranded DNA virus (> 8 kb), that replicates in the nucleus of infected host cells. Until now, more than 100 HPV genotypes have been identified and the ratio of HPV positive EP ranges from 10.5 to 21.3% [10, 20]. In the reports of 53 EPS cases, 29 cases underwent HPV detection, 37.9% (11/29) detected HPV in these patients (Table 1). HPV testing methods vary from immunohistochemistry (IHC), dot blot hybridization (DB), in situ hybridization (ISH), polymerase chain reaction (PCR) to transmission electron microscopy (TEM). Some researchers suggested that PCR method should be used to test for HPV DNA due to its high sensitivity [10, 29]; however, others thought that even with more sensitive molecular analysis techniques, HPV DNA is not consistently recovered from the lesions [30, 31]. Thus, the true incidence of HPV infection may be underestimated, and the main reason may be due to the currently available HPV testing methods that only detect the most common viral subtypes.

**Table 1:** EPS Cases with HPV infection

Source	Age	Sex	Esophageal involvement	HPV type	Assay technique	With extraesophageal papillamas	With esophageal cancer
Janson et al. [21]	35	M	Middle	6,11	DNA probe	–	−
Politoske et al. [22]	37	M	Middle	6, 11	ISH	–	−
Batra et al. [23]	2	F	Cervical	6, 11	ISH	RRP (Supraglottis, Glottis)	−
Mészner et al. [7]	3.5 m	M	Whole	6, 11	molecular biological methods	RRP (Larynx, Hypopharynx)	−
Sarita Singhal et al. [24]	2	M	Whole	6, 11, 42, 43, 44	ISH	Pharynx	−
						Uvula	
						Larynx	
Hording et al. [25]	27	M	Distal	11	DB	Bronchial papillomatosis	−
						Skin warts	
Saravana et al. [15]	60	M	Distal	5, 16	PCR	–	+
Van Cutsem et al. [26]	69	F	Middle	16, 18	PCR	Hypopharynx	+
Kato et al. [27]	83	M	Whole	16, 33	PCR	–	−
Romano et al. [28]	53	F	Whole	16	ISB	–	−
Ravakhah et al. [29]	53	F	Whole	51, 52, 56	PCR	Condyloma acuminate of the vulva and perineum	−

*ISH* in situ hybridization, *DB* dot-blot hybridization, *PCR* polymerase chain reaction, *RRP* recurrent respiratory papillomatosis

In 11 HPV positive cases, 12 virus types were detected (Table 1) and approximatively half of these (6/11) harbored HPV types 6 and/or 11. It is well known that the vast majority of recurrent respiratory papillomatosis (RRP) lesions are caused by the above 2 types [32]. It's worth mentioning that 4 of these were associated with respiratory papillomas, with an average age not exceeding 7.8-year-old [7, 23, 24]. We can speculate that to some extent, EPS can co-exist with the respiratory system papillomas when HPV positive. HPV can be grouped as high or low risk according to their contribution to malignant changes. HPV types 6 and 11 belongs to the low-risk type that may induce benign tumors. According to the reports, 4 cases (4/11) involved HPV type 16, which is considered as a high-risk type that is strongly associated with malignancies. And half of the cases suffered from esophageal cancer [15, 26]. When a high-risk type HPV is detected, we need to be aware of malignant tumors.

Although HPV plays an important role in the occurrence of EPS, it is not yet clear how patients get infected. Morris [33] proposed that when newborns passage through an HPV infected canal, the transmission may occur in the upper respiratory/digestive tracts by contamination. In the EPS reports, delivery [6], sexual [29], and direct contact transmission [24] may facilitate HPV implantation.

### Genetic mutation

In addition to chronic mucosal irritation and HPV infection, genetic factors are also involved in EPS pathogenesis. Among the 53 cases, 6 Focal dermal hypoplasia (FDH), also been called Goltz syndrome, were reported to manifest with EPS (Table 2). Goltz syndrome is a rare multisystem disorder that is characterized by cutaneous, skeletal, dental, ocular, and soft-tissue defects. Researchers suppose that Goltz syndrome is caused by genetic mutations in the PORCN gene [40, 41] that is associated with a X-linked dominant condition, characteristic of female genetic predominance. The 6 cases were all females aged between 3 and 56 years old. Two of them were proved to carry a PORCH gene mutation. Nasr [38] proposed that EPS could be the gastrointestinal phenotypic manifestation of Goltz syndrome. In a four-generation family with angioma serpiginosum (AS), among the six female patients, two sisters of the third generation had EPS [42]. AS is a congenital nevoid disorder, characterized by pinpoint violaceous to coppery red punctuates on an erythematous base. Both autosomal and X-linked dominant inheritances have been considered. In this family, an X-linked dominant condition mapping to Xp11.3-Xq12 was confirmed. In conclusion, genetic mutations may be related to EPS development.

**Table 2:** Goltz syndrome cases with EPS

Source	Age	Sex	Esophageal involvement	Digestive symptoms and signs	Typical manifestations	Genetic mutations
Brinson et al. [34]	30	F	Distal	Dysphagia	Dermatitis	N
				GERD	Sclerodactyly	
				Esophageal stricture	Hyperpigmentation	
				Hiatus hernia	Dysplasia of skeleton, teeth	
Kashyap et al. [35]	56	F	Distal	Dysphagia	Erythematous dermatitis	PORCN
				Heartburn	Syndactyly	
				GERD	Split nails	
Helga Bertani et al. [36]	33	F	Middle and distal	Dysphagia	N	N
				Chronic reflux disease		
Pasman et al. [37]	8	F	Distal	Dysphagia	Scars	N
				Lose weight	Excoriated facial rash	
				Eosinophilic esophagitis	Dysmorphic facies	
					Cleft lip and palate	
Nasr et al. [38]	3	F	Whole	Gastric ulcer	Parakeratosis	PORCN
				Chronic iron deficiency anemia	Dysmorphic facies	
					Cleft lip and palate	
					Polydactyly	
					Right microphthalmia	
Hafiz et al. [39]	37	F	Distal	Dysphagia	Oligodactyly	N
				Poor appetite	Lobster-claw	
				Weight loss	Hypopigmentation	
				Anemia	Hyperpigmentation	
				SCC		

*GERD* gastroesophageal reflux disease, *SCC* esophageal squamous cell carcinoma, *N* not mention

From the series of EPS cases, we cannot rule out the possibility that EPS may be caused by the cooperation of multiple factors.

## Clinical manifestation of EPS

### Symptoms

In contrast to the clinical limitations associated with the isolated papilloma, EPS shows several clinical symptoms. Dysphagia is the first observed symptom and constitutes the most typical manifestation of this condition. Over half of the EPS patients (32/53) bore this pain regardless of age that ranges from 2 to 84 years old [24, 43]. Heartburn is less common than dysphagia and some patients have been diagnosed as gastroesophageal reflux disease (GERD) or chronic reflux disease. Other symptoms, such as epigastric abdominal discomfort and dyspepsia, were occasionally reported. Other patients also presented with respiratory symptoms when associated with respiratory papillomatosis, such as noisy breathing, stridor and aphonia [6, 7, 23, 24, 44]. More than one in six patients lost weight and some asymptomatic patients were accidentally discovered [8, 45–48].

### Endoscopic manifestations

**Fig. 1:**
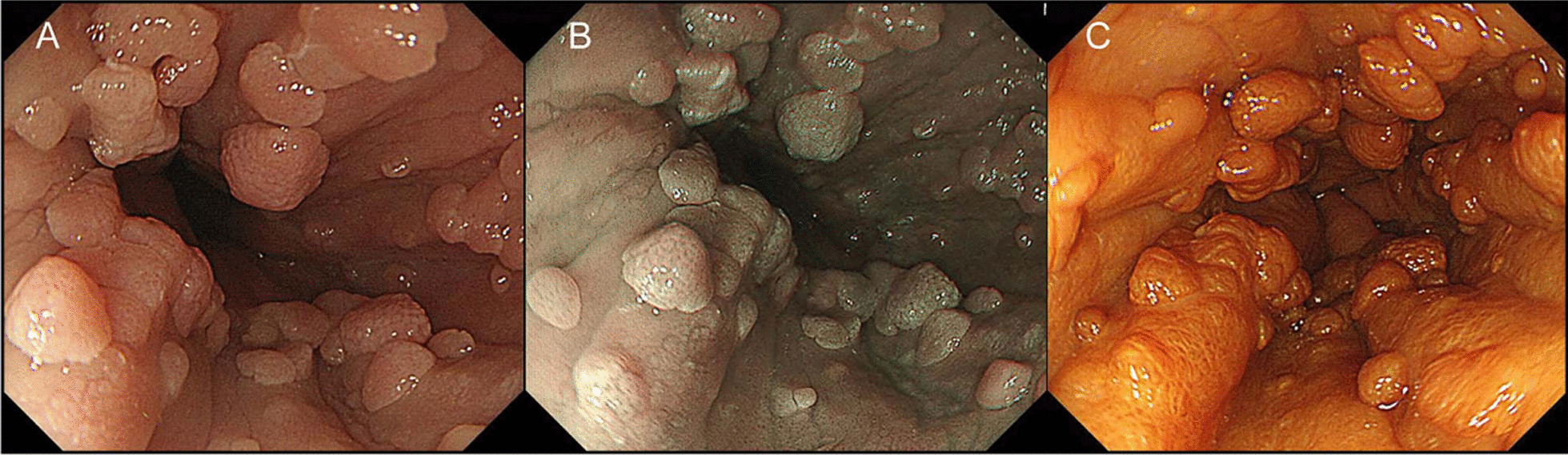
Esophageal endoscopy with **A** white light, **B** narrow-band imaging, and **C** Lugol spraying. Many elevated lesions were present in the esophagus (predominantly at the distal esophagus) deeper than the dental arch at 25 cm, without abnormal blood vessels on narrow-band imaging and a stainless band by Lugol spraying (Makise et al. [47])

Although some cases were first identified by esophagogram, which showed the presence of multiple filling defects, the diagnosis of EPS has to rely on esophagogastroduodenoscopy (EGD). The lesions usually appear as small, white or pink colored, with a smooth or slightly rough surface, sessile or pedunculated forms and with a close-set pattern (Fig. 1) [47]. Because of the extending growth pattern, papillomas are uncountable. It can grow as limited or widespread and with variable sizes, ranging from 1 to 15 mm [7, 49]. A total of 21 cases showed that the papillomatosis spread throughout the whole esophagus. Others range from 2 to 12 cm and most of the lesions covered the middle and/or distal esophagus. In addition, varying degrees of esophageal strictures can be found. It is noteworthy to point out that all lesions of the Goltz syndrome with EPS, involved the distal esophagus (Table 2). Special endoscopy technologies were also used to detect and distinguish EPS lesions, such as narrow band imaging (NBI) with magnifying endoscopy [45, 47, 50], and Lugol’s solution staining [14, 51] and endoscopic ultrasonography [39, 52, 53].

### EPS cases complicated with esophageal cancer

EP was classified as an esophageal benign epithelial tumor and its association with cancer is extremely rare [54, 55]. Although only a handful of EPS cases were disclosed, 12 cases were reported to be complicated with esophageal cancer, accounting for approximatively 22.6% of all cases (Table 3). There are no differences between female and male cases and half of the lesions were in the middle esophagus. The pathology suggested the presence of an esophageal squamous cell carcinoma (ESCC), which development is frequently noted in HPV associated diseases. However, only 2 cases, that had an HPV infection, were confirmed (Table 2). It seems that in this situation, HPV is not the main factor inducing ESCC. Additionally, high-grade dysplasia can be detected in the EPS tissue [16, 28, 60]. Interestingly, some patients acquired EPS, 1–3 year after esophageal carcinoma esophagectomy and gastric carcinoma surgery [17, 49, 58, 61], in which gastroesophageal reflux may play an important role during the process. Other EPS cases can also be found to be associated with oropharyngeal cancer and gastric adenocarcinoma [27, 62, 63], for which there are no suitable explanations. Thus, the cause of the malignant transformation is unclear and controversial; however, the malignant potential of EPS cannot be ignored.

**Table 3:** EPS cases complicated with esophageal cancer

Source	Age	Sex	Esophageal cancer involvement	Pathology	Therapy
Van Cutsem et al. [26]	69	F	Middle	Dysplasia and invasive epithelial cell nests	Esophagectomy Nd-Yag laser Photocoagulation Alpha-interferon treatment HPMPC local injection
Waluga et al. [51]	28	M	Proximal	Poor-differentiated ESCC	N
Wolfsen et al. [43]	84	M	Distal	Microinvasive well-differentiated ESCC	PDT Metal stent placement
Reynoso et al. [56]	74	F	Middle	In-situ ESCC with microinvasion into the lamina propria	Esophagectomy
Kao et al. [52]	70	F	Middle	Invasive ESCC	Repeated dilatation Metal stent placement
Saravanan et al. [15]	60	M	Distal	Invasive well-differentiated ESCC with dysplasia	Esophagectomy
Attila et al. [57]	70	M	Middle	Invasive well-differentiated ESCC	EMR Esophagectomy
Donnellan et al. [48]	64	F	Middle	Invasive ESCC	Chemoradiotherapy
Pohl [58]	72	F	Middle	ESCC	Esophagectomy APC endoscopic mucosectomy
Tanimu et al. [53]	51	M	Distal	ESCC	EMR, APT, PDT, Radiofrequency ablation Esophagectomy
Bronswijk et al. [59]	47	M	Proximal	ESCC	Radiochemotherapy
Hafiz et al. [39]	37	F	Distal	Moderately differentiated invasive ESCC	Chemoradiotherapy Esophagectomy

*PDT* photodynamic therapy, *ESCC* esophageal squamous cell carcinoma, *EMR* endoscopic mucosal resection, *APC* argon plasmacoagulation, *HPMPC* (S)-l-(3-hydroxy-2-phosphonylmethoxypropy1)cytosine, *N* not mention

The diagnosis of EPS with ESCC is extremely difficult and time-consuming due to the extensive and numerous lesions. To ensure a better results’ accuracy, some patients had to undergo multiple biopsies (from 2 to 15 times) and endoscopic mucosal resection (EMR) [52, 53, 56, 57]. Moreover, it took the clinicians 3–9 years to establish a diagnosis, and in some cases, the confirmation required esophagectomy [53, 56, 57]. Therefore, when EPS is disclosed, accurate and repeated biopsies are necessary.

### EPS cases complicated with extraesophageal papillomas

From the reports, 13 of the 53 cases were complicated with extraesophageal papillomas (Table 4). Interestingly, 84.62% (11/13) of the lesions were located at the respiratory tract. Mucocutaneous papillomas are a common finding of the Goltz syndrome, and EPS is a rare manifestation (Table 2). This also happens with acanthosis nigricans (AN) [45], a condition, characterized by a dark pigmented thickening of the skin, with hyperkeratosis and papillomatosis and a basal layer hyperpigmentation. Patients with RRP can also associate with EPS, which is HPV induced. Therefore, we should pay more attention to patients with respiratory papillomas, whether affected with Goltz syndrome and AN, or with HPV infection.

**Table 4:** EPS cases complicated with extraesophageal papilloma

Source	Age	Sex	Extraesophageal papilloma	Non-digestive symptom	Special diagnosis	HPV type
Nasr et al. [38]	3	F	Eyelids Oropharynx	Upper respiratory infection	Goltz syndrome	–
Kashyap et al. [35]	56	F	Oral cavity Axilla Inguina	–	Goltz syndrome	–
Hafiz et al. [39]	37	F	Face Lip	–	Goltz syndrome	–
Amano et al. [45]	60	M	Pharynx Anal canal	–	AN	–
Batra et al. [23]	2	F	Supraglottis Glottis	Nocturnal stridor Hoarseness	RRP	6, 11
Mészner et al. [7]	3.5 m	M	Larynx Hypopharynx	Aphonia Stridor	RRP	6, 11
Singhal et al. [24]	2	M	Pharynx Uvula larynx	Noisy breathing	–	6, 11, 42, 43, 44
Hording et al. [25]	27	M	Bronchia Hand warts	Periodical subfebrility Hacking cough	–	11
Ravakhah et al. [29]	53	F	Vulva Perineum	–	Condyloma acuminata	51, 52, 56
Nuwayhid et al. [6]	2.5	M	Hypopharynx	Stridor Respiratory difficulty	Retroperitoneal neuroblastoma Mother with vulvar condylomata	–
Van Cutsem et al. [26]	69	F	Hypopharynx	–	–	16, 18
Frootko et al. [44]	6	F	Hypopharynx	Stridor Hoarseness	–	–
Sandvik et al. [64]	28	F	Pharynx	–	–	–

*RRP* recurrent respiratory papillomatosis, *AN* canthosis nigricans

## Histopathology of EPS

**Fig. 2:**
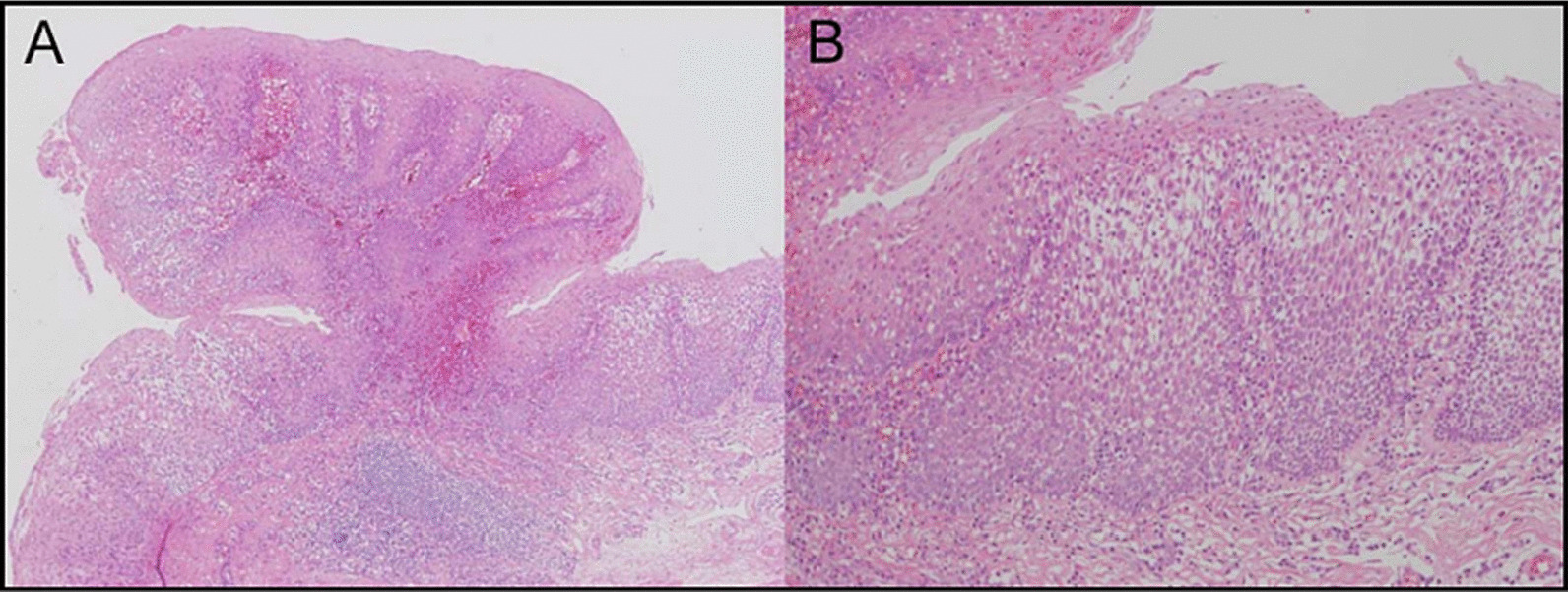
Hematoxylin and eosin staining of the pathological specimens. **A** The subsidiary raised area was composed of irregularly thickened stratified squamous epithelium and a core of fibrovascular tissue. **B** The surface of the lesions was relatively smooth, and thick rete-like epithelial projections extended in the stromal core (Makise et al. [47])

Although EPS is characteristic under endoscopic examination, its diagnosis should meet the pathological diagnosis. The most typical appearance is the fibrovascular core and numerous finger-like projections that are covered with hyperplastic squamous epithelium (Fig. 2) [47]. The appearance of koilocytes, expressing hyperchromatic nuclei and perinuclear halos, supports an HPV squamous epithelium infection. It can be seen in nearly half of the HPV positive cases with EPS [15, 21, 24, 26, 27]. However, although the above histologic findings can be disclosed, it does not mean that HPV could be detected [64]. Until now, all the histopathological diagnoses of an EPS invasive malignancy showed ESCC (Table 3). Different degrees of dysplasia can also be discovered [14, 25, 28, 60]. Some reports suggested that EPS should be considered as a premalignant lesion due to its ESCC potential [15, 48]. The presence of inflammation is not unusual in the diagnosis of EPS lesions, and the existence of eosinophilic infiltration can also be disclosed in some cases [37, 47, 53, 57].

## Treatment of EPS

EPS treatment is controversial and extremely challenging due to the extensive nature of the lesions and the repeated recurrence. Although faced with variable rates of failure, clinicians are exploring different treatment methods. EPS current managements are mainly divided into medical, endoscopic, and surgical therapy.

### Medicine treatment

To improve the symptoms of gastroesophageal reflux, anti-reflux medications, such as proton pump inhibitors (PPI) are commonly used. Since HPV infection was suggested as one of the EPS causes, etiological treatments were therefore, administered. For instance, a 3.5-month-old boy suffering from EPS and RRP, with HPV type 6 and 11 infections, underwent a total of 11 repeated microlaryngoscopic surgeries and laser photocoagulation. Unfortunately, the treatment effects were poor and resulted in a repeated recurrence [7]. When he was 2 1/2-year-old, he was treated with a 4-valent HPV vaccine and after three doses, both laryngeal and esophageal lesions completely disappeared and no papillomas were detected after 2 years follow-up. Interferon alpha can also be used to treat the disease based on its antiviral effects [25, 26]. However, it seems that it did not work well during the process. It is worth mentioning that a case of treatment with (S)-l-(3-hydroxy-2-phosphonylmethoxypropy1) cytosine (HPMPC) was successful [26]. The patient suffered recurrent EPS attacks for approximatively 6 years and during that period, she unsuccessfully received various treatments, including esophagectomy, Nd–YAG laser photocoagulation and interferon alpha injection, until HPMPC was introduced. Local injections of HPMPC were performed 10 times during the 7 months following the lesions’ gradual regression. The reporter speculated that one of the potential mechanisms was the inhibitory effect of the HPMPC diphosphate on the viral DNA polymerization process.

### Endoscopic treatment

Various endoscopic treatments were used to manage the EPS lesions, including argon plasma coagulation (APC), Nd-YAG laser, radiofrequency ablation, cryotherapy and photodynamic therapy (PDT). The first three types induce rapid coagulative necrosis in EPS tissues through the heating effect. Although part of the effects was temporary, they were confirmed to be helpful in clearing away the lesions [25, 26, 36, 37, 58, 65]. Cryotherapy with spray of liquid nitrogen cryogen under endoscopy was successfully introduced to remove EPS [62, 66], which led lesions’ degeneration and necrosis. Additionally, Wolfsen et al. [43] were the first to introduce PDT to the therapy of EPS and provided a successful example. Porfimer sodium was used as photosensitizer that generates reactive oxygen and induced mucosal necrosis following laser radiation. Furthermore, EMR can be used when the lesion is limited [58, 67] and no previous studies attempted resecting the lesion by endoscopic submucosal dissection (ESD). Perhaps ESD is another viable option since it can remove a wide range of mucosa. Although the above treatments raised hopes, some studies considered that endoscopic therapy was not amenable for this condition [50, 53] due to the extensiveness of the lesions. Despite the effectiveness of these treatments in the short term, endoscopists always felt helpless against EPS repeated recurrence. In addition, to improve the esophageal stricture, repeated dilatations, and metal stent placements, were also adopted [14–16, 43, 52].

### Surgery

Esophagectomy is the most ideal way to eliminate EPS with ESCC [15, 26, 39, 53, 56–58] and this treatment was also used to treat patients with severe dysplasia [28, 60]. The surgical options vary from subtotal to total esophagectomy. Most lesions were removed by operation despite some small, remaining, and recurrent papillomatosis in the residual esophagus and the anastomotic site, which can be cleared away by endoscopic management. However, the choice of surgery is still prudent due to the cases that are mostly benign.

Among the 30 cases that received the above treatments, only 9 (30%) had been reported to be recurrence free during the 3 months to 2 years follow-up. Raising an optimal management is extremely difficult for clinicians since no guidelines can be followed. Except for esophagectomy, the medicine and endoscopic treatments are shown in Table 5.

**Table 5:** The medicine and endoscopic treatments for EPS

	Source	Methods	Outcome
*Medicine*			
PPI	Pasman et al. [37]	N	Inefficacy
	Gencdal et al. [68]	N	Improvement of symptoms
Alpha-interferon	Hording et al. [25]	4 MIU daily, subcutaneously for 4 weeks, double dose for 2 weeks, 8 MIU three times weekly	Early improvement and subsequent recurrence
	Van Cutsem et al. [26]	6 × 10^6^ U five times a week for 4 weeks followed by three times a week for 2 months	Recurrence
Vacine	Mészner et al. [7]	Three doses of the quadrivalent HPV vaccine at 0, 2 and 6 months	Elimination
*Endoscopy*			
APC	Pasman et al. [37]	ERBE VIO APC system,with an effect of 5	Elimination
Radiofrequency ablation	Romano et al. [28]	The first procedure treatment: the 360° catheter; the second and third treatment: the Barrx Channel catheter at 12 J/cm^2^	Elimination
Photodynamic therapy	Wolfsen et al. [43]	Infusion of porfimer sodium (2 mg/kg), with endoscopic delivery of 300–400 J/cm fifiber length red light energy (wavelength 630 nm)	Remission
Cryotherapy	McDonald et al. [62]	Liquid nitrogen cryotherapy	Elimination
	Alomari et al. [66]	Liquid nitrogen cryogen applied for 20 s, 3 sessions, 2–4 months apart	Elimination
Laser therapy	Hording et al. [25]	CO_2_-laser: 4 times	Inefficacy
	Tu et al. [65]	Nd:YAG:50 watts, 0.2 s duration, and 0.1 s intervals	Elimination
Local injections of HPMPC cytosine	Van Cutsem et al. [26]	HPMPC 1.25 mg/kg, the first four injections: an interval of 1 week, the next three injections: an interval of 3–5 weeks	Elimination
EMR	Kim et al. [67]	Performed by a band mucosectomy device	Elimination
Dilation	Narayani et al. [14]	Progressive dilation	Temporary improvement and subsequent progression
	Saravanan et al. [15]	Repeated dilation	
	Kao et al. [52]	Repeated dilation	

*PPI* proton pump inhibitor, *APC* argon plasma coagulation, *EMR* endoscopic mucosal resection, *HPMPC* (S)-l-(3-hydroxy-2-phosphonylmethoxypropy1)cytosine, *N* not mention

## Clinical course of EPS

The natural history of EPS remains unclear and from the literature, we found that the EPS clinical course varies from spontaneous regression to the development of ESCC. Some researchers recommended that EP should be removed because of the potential malignancy [69]. Nevertheless, is it necessary to provide positive treatments to each patient suffering from EPS? Some reports gave different puzzling ideas.

Among all the 53 cases, 4 cases presented a spontaneous regression of EPS during 1 month to 3.5 years and without active treatments that target the esophageal lesions [24, 27, 44, 64]. HPV was detected in 2 cases [24, 27] and in another case koilocytes were found following histological examination [64], which implying a relative HPV-related regression. A similar regression can also be seen in cervical diseases associated with HPV infections [70]. Kato et al. [27] presented a special case of complete EPS regression, only 1 month after total gastrectomy, due to gastric cancer. They considered that the end of the HPV infection and the remission from acid reflux, promoted the regression.

Furthermore, the clinical course can express a stable process while, a proportion of the EPS lesions had no development in the following 1–7 years and therefore, no treatments were undertaken [22, 23, 45, 47, 50]. Of course, the most common course is a slow progression. Hording et al. [25] presented a rapid and fatal course, where a 27-year-old man underwent EPS and bronchopulmonary papillomatosis both harboring HPV11, and who unfortunately died 1 year and 10 months later, due to an acute rupture of an eroded vessel. Therefore, with variations in the clinical course, close endoscopic surveillance is necessary for early detection of the malignant lesions.

## Conclusions

EPS is an extremely rare disease that has no standard therapeutic or surveillance guidelines. Based on the existing literature, we have a certain knowledge of EPS.

1. Clinicians should be alert when respiratory papillomatosis or Goltz syndrome is disclosed, especially along with dysphasia symptoms.
2. EPS diagnosis relies on the combination of endoscopic performances and pathological features.
3. Once the diagnosis is established, an HPV test should be performed to estimate the risk level and explore potential therapeutic strategies.
4. EPS is a benign lesion with a highly malignant potential.
5. The aim of available therapies is to control or treat EPS-associated symptoms. Esophagectomy is necessary when ESCC is confirmed; however, recurrence remains the biggest problem.
6. Continuous endoscopic surveillance, with careful and multiple biopsies, is necessary due to the progressive clinical course of most EPS cases and the potential for malignant degeneration.

## Data Availability

Not applicable.

## Abbreviations

EP: Esophageal papilloma
EPS: Esophageal papillomatosis
HPV: Human papilloma virus
DEN: Diethylnitrosamine
IHC: Immunohistochemistry
DB: Dot blot hybridization
ISH: In situ hybridization
PCR: Polymerase chain reaction
TEM: Transmission electron microscopy
RRP: Recurrent respiratory papillomatosis
FDH: Focal dermal hypoplasia
AS: Angioma serpiginosum
GERD: Gastroesophageal reflux disease
EGD: Esophagogastroduodenoscopy
NBI: Narrow band imaging
ESCC: Esophageal squamous cell carcinoma
AN: Acanthosis nigricans
PPI: Proton pump inhibitors
HPMPC: (S)-l-(3-hydroxy-2-phosphonylmethoxypropy1) cytosine
APC: Argon plasma coagulation
PDT: Photodynamic therapy
ESD: Endoscopic submucosal dissection
